# Absence of Relationship Between Self-Reported Sleep Measures and Amyloid Load in Elderly Subjects

**DOI:** 10.3389/fneur.2019.00989

**Published:** 2019-09-13

**Authors:** Audrey Gabelle, Laure-Anne Gutierrez, Isabelle Jaussent, Fayçal Ben Bouallegue, Delphine De Verbizier, Sophie Navucet, Caroline Grasselli, Karim Bennys, Cécilia Marelli, Renaud David, Denis Mariano-Goulart, Sandrine Andrieu, Bruno Vellas, Pierre Payoux, Claudine Berr, Yves Dauvilliers

**Affiliations:** ^1^Department of Neurology, Memory Research and Resources Center, CHU Montpellier, Montpellier, France; ^2^INSERM, U1061, Neuropsychiatrie, Recherche Clinique et Epidémiologique, Montpellier, France; ^3^Université de Montpellier, MUSE, Montpellier, France; ^4^Nuclear Medicine Department, CHU Montpellier, Montpellier, France; ^5^PhyMedExp, INSERM-CNRS, Montpellier University, Montpellier, France; ^6^Department of Psychiatry, Memory Research and Resources Center, CHU Nice, Nice, France; ^7^Gérontopôle de Toulouse, Inserm UMR1027, Toulouse Université III, Toulouse, France; ^8^Nuclear Medicine Department, CHU Toulouse, Toulouse, France; ^9^Department of Neurology, Narcolepsy National Reference Center, Sleep Center, CHU Montpellier, Montpellier, France

**Keywords:** amyloid, amyloidosis, sleep, elderly, PET—positron emission tomography, NAPS, dementia, cognition

## Abstract

**Objective:** To determine the relationships between self-reported sleep profile and cortical amyloid load in elderly subjects without dementia.

**Methods:** This cross-sectional study included 143 community-dwelling participants aged ≥70 years (median: 73 years [70–85]; 87 females) with spontaneous memory complaints but dementia-free. Sociodemographic characteristics, health status, neuropsychological tests, sleep, and ^18^F-florbetapir (amyloid) PET data were collected. The clinical sleep interview evaluated nighttime sleep duration, but also daytime sleep duration, presence of naps, and restless leg syndrome (RLS) at time of study. Validated questionnaires assessed daytime sleepiness, insomnia, and risk of sleep apnea. The cortical standardized uptake value ratio (SUVr) was computed across six cortical regions. The relationship between sleep parameters and SUVr (cut-off ratio>1.17 and tertiles) was analyzed using logistic regression models.

**Results:** Amyloid-PET was positive in 40.6% of participants. Almost 40% were at risk for apnea, 13.5% had RLS, 35.5% insomnia symptoms, 22.1% daytime sleepiness, and 18.8% took sleep drugs. No significant relationship was found between positive amyloid PET and nighttime sleep duration (as a continuous variable, or categorized into <6; 6–7; ≥7 h per night). Logistic regression models did not show any association between SUVr and daytime sleep duration, 24-h sleep duration, naps, RLS, daytime sleepiness, insomnia symptoms, and sleep apnea risk (before and after adjustment for APOEε4 and depressive symptoms).

**Conclusion:** Our study did not confirm the association between amyloid-PET burden, poor sleep quantity/quality in elderly population, suggesting that the interplay between sleep, and amyloid is more complex than described.

## Introduction

Experimental and human studies suggest that sleep-wake alterations contribute to brain amyloid-beta (Aβ) dysregulation and showed that Aβ load in interstitial fluid proportionally increases with time awake (1). Short sleep duration, sleep fragmentation, and reduced slow-wave sleep may thus affect Aβ brain deposition, one of the key pathophysiological mechanisms of Alzheimer's disease (AD) (1–3). Sleep-wake disturbances (i.e., sleep fragmentation, frequent and long awakenings, and excessive daytime sleepiness), and sleep disorders (i.e., insomnia and sleep apnea syndrome) are frequently found in patients with AD at early and also late stages of the disease (4–10).

One reference study on 62 older adults found that people with self-reported short sleep had higher Aβ burden than long sleep/night (5). Other studies on cognitively healthy individuals at risk of developing AD reported associations between amyloid load and other sleep parameters (i.e., sleep quality, sleep latency, sleep efficiency, wake after sleep onset, excessive daytime sleepiness, and napping) (5–8, 11–14). Similarly, in a 1-year prospective study, we found that the risk of cognitive decline is higher in frail elderly subjects with excessive daytime sleepiness and longer nighttime in bed (15). However, all these results need to be replicated due to the frequent sleep misperception in older people, and the differences in sleep assessment methodology, populations and sample size, treatment intake, confounding factors and study design (5–14). Moreover, the relationships between sleep profile, cognitive status, and amyloid load measured by positron imaging tomography (PET) remain unknown in elderly population with memory complaints.

The main aim of this study was to determine the relationship between self-reported nighttime sleep duration and cortical amyloid load, measured by PET with the ^18^F-florbetapir amyloid ligand (^18^F-florbetapir PET), in elderly subjects with memory complaints. We also assessed using a detailed, and comprehensive clinical interview the relationships between other sleep characteristics and brain amyloid load.

## Methods

### Participants

The MAPT-AV45 sleep ancillary study (www.clinicaltrials.gov NCT00672685) included 143 community-dwelling participants aged ≥70 years (median age: 73 years-old; range [70 to 85]; 87 females) with spontaneous memory complaints, but free of dementia (16–19). Sociodemographic characteristics, health status, and neuropsychological test results were available for all patients who also underwent sleep assessment and ^18^F-florbetapir-PET brain imaging.

The MAPT-AV45 study and the sleep ancillary study were approved by the Toulouse ethics committee (Comité de Protection des Personnes- Sud-Ouest et Outre-Mer I et II). The methods were carried out in accordance with the approved guidelines. Each participant signed legal consent forms. Informed consent was obtained from all subjects.

### ^18^F-florbetapir-PET Brain Imaging

Brain imaging was performed using five different hybrid PET-CT scanners. Image acquisition started 50 min after injection of 4 MBq/kg of ^18^F-florbetapir. PET sinograms were reconstructed with an iterative algorithm, with corrections for randomness, scatter, photon attenuation, and decay, producing images with an isotropic voxel of 2 × 2 × 2 mm^3^, and a spatial resolution of approximately 5-mm full width at half maximum at the field of view center. ^18^F-florbetapir images were co-registered to a template provided by Avid Radiopharmaceuticals (Philadelphia, PA) (20) using SPM for normalization to the Montreal Neurological Institute (MNI) space. Tracer retention in the cerebral cortex was quantified using the standardized uptake value ratio (SUVr) relative to the whole cerebellum. The SUV of the cortical retention index were computed in six cortical regions of interest (i.e., frontal, parietal, temporal, precuneus, anterior, and posterior cingulate cortices). Cortical SUV ratios were obtained by normalizing the cortical SUV with the mean uptake relative to the whole cerebellum that defines the amyloid load. According to a previously established cut-off, the amyloid PET scan was considered positive when the global cortical SUVr value was higher than 1.17^3^. As threshold may depend of the targeted population, we further grouped participants according to the tertiles of their global cortical SUVr, the highest tertile group compared with the other two.

### Self-Reported Sleep Parameters

All participants had a face-to-face interview to assess last month the duration of nighttime sleep (in hour and also categorized as <6; 6–7; ≥7 h per night according to a reference study ^10^), daytime sleep (in minutes), presence and duration of naps (recorded as: no naps, naps <30 min, naps ≥30 min per day), total sleep time (daytime and nighttime sleep), and sleep efficiency (i.e., total sleep time divided by time spent in bed reported by subjects, expressed as a percentage). The presence of restless legs syndrome [RLS; on the basis of the five minimal diagnostic criteria (21)], and of rapid eye movement sleep behavioral disorder (RBD; violent nocturnal agitation accompanied by shouting often at the end of the night and associated with dreamlike memories) were also investigated by a semi-structured clinical interview. Participants completed, with the help of medical staff when required, the following sleep questionnaires: the Epworth Sleepiness Scale (ESS) to evaluate excessive daytime sleepiness (EDS; cut-off score ≥11) (22); the 7-item self-report Insomnia Severity Index (ISI) to evaluate insomnia symptoms [score <8: low insomnia; score (3–9): sub-threshold insomnia; and score ≥15: moderate-severe insomnia] (23); and the Berlin questionnaire to assess the risk of sleep apnea (24).

### Other Biological and Clinical Characteristics

A standardized interview with questions on sociodemographic characteristics, health status and use of medications was performed at baseline and each year during the 5-year-follow-up. Drugs were coded according to the World Health Organization's Anatomical Therapeutic Chemical Classification. Hypnotics were classified as benzodiazepine, benzodiazepine-like compounds (zolpidem, zopiclone), sedative antidepressants, and miscellaneous medications (including barbiturates, antihistamines, and other pharmacological categories, such as neuroleptics). Drugs administered during the year of the ^18^F-florbetapir-PET were taken into account for the analysis. Height and weight were measured and used to calculate the body mass index. Cerebro-cardiovascular and metabolic diseases were defined as self-report history of stroke and cardiovascular events, diabetes, or hypertension (defined by measured systolic blood pressure ≥140 mmHg or diastolic blood pressure ≥95 mmHg, or current antihypertensive treatment). Depressive symptoms were evaluated using the Beck Depression Inventory II (BDI-II) scale (25) (no depressive symptoms: 0–11 score; moderate to severe depressive symptoms, when score ≥12).

Based on the Clinical Dementia Rating (CDR) scale and the validated Petersen criteria (26), participants with a CDR score = 0.5 were classified as having Mild Cognitive Impairment (MCI). Participants with dementia at baseline (CDR score ≥ 1) were not included. The Mini Mental State Examination (MMSE) test was performed in all participants.

APOE was genotyped using a classical PCR digestion method (primers: 5′-GGGCACGGCTGTCCAAGGAGCTG-3′ and 5′-TCGCGGGCCCCGGCCTGGTA CACT-3′; restriction enzyme: *Hha*1).

### Statistical Analysis

The sample was described using percentages for categorical variables, and medians [range] for quantitative variables, because the Shapiro-Wilk test indicated the data distribution was mostly skewed. The negative and positive amyloid PET groups were compared using univariate logistic regression models. Sociodemographic and clinical variables associated with brain amyloid load at *p* < 0.10 in the univariate analysis were included in logistic models to estimate the adjusted odds-ratios (OR) and their 95% confidence intervals (CI) for sleep parameters. For all analyses, the significance level was set at *p* < 0.01. Analyses were performed using the SAS statistical software (version 9.4; SAS Inc, Cary, North Carolina).

## Results

### Clinical Characteristics, Sleep Profile, and Amyloid PET

For the whole population (*n* = 143), the education level was intermediate in 45.5% and high in 33.6% of participants, and the median MMSE score was 29 [20–30]. Moreover, 25% of participants had MCI, and 28.5% were APOEε4 allele carriers. Regarding the vascular and metabolic profile, 41.3% were overweight, 17.5% obese, and 65.7% reported history of cardiovascular and/or metabolic diseases. Moderate to severe depressive symptoms were reported by 30.0% of participants.

The median sleep duration was 7 h [3.25–9.5] for nighttime and 20 min [0–120] for daytime. About 56% of participants were nappers, and 29.5% had naps longer than 30 min. The median sleep efficiency was 87.5% [44.4–100]. Almost 40% of participants were at risk of sleep apnea, 13.5% had RLS (mild in 52.6% of them), 22.1% had EDS, 35.5% presented insomnia symptoms (moderate to severe for 8.5% of them), 8.2% had clinically-defined RBD, and 18.8% took often hypnotics.

Amyloid PET was positive (global cortical SUVr >1.17) in 40.6% of participants (Table 1). Participants with positive amyloid PET were more frequently APOEε4 carriers (*p* = 0.003) and had more often depressive symptoms (BDI-II score ≥12) (*p* = 0.03) (Table 1).

**Table 1:** Socio-demographic and clinical characteristics of the participants divided according the pathological cut-off value for the global cortical SUVr calculated using ^18^F-florbetapir-PET data.

**Variable**	**Global cortical SUVr**				***p***
	**≤1.17** ***N*** **=** **85**		**>1.17** ***N*** **=** **58**		
	***N***	**%**	***n***	**%**	
**Sex**					
Men	55	64.71	32	55.17	0.25
Women	30	35.29	26	44.83	
**Age, in years**^**(a)**^	74 [70–85]		73 [70–85]		0.86
**Educational level**					
Low	22	25.88	8	13.79	0.20
Intermediate	35	41.18	30	51.72	
High	28	32.94	20	34.48	
**MMSE score**^**(a)**^	29 [24–30]		29 [20–30]		0.45
**MCI**					
No	65	76.47	42	72.41	0.58
Yes	20	23.53	16	27.59	
**APOEε4**					
Not carrier	62	81.58	31	57.41	**0.003**
Carrier	14	18.42	23	42.59	
**BMI, kg/m2**					
<25	33	38.82	26	44.83	0.75
[25–30]	36	42.35	23	39.66	
≥30	16	18.82	9	15.52	
**BDI-II score**					
<12	67	78.82	35	61.40	**0.03**
≥12	18	21.18	22	38.60	
**Cardiovascular events**					
No	29	34.12	20	34.48	0.96
Yes	56	65.88	38	65.52	
**Hypnotic intake**					
No	70	82.35	46	79.31	0.65
Yes	15	17.65	12	20.69	

(a) *Continuous variables were expressed as median [minimal value–maximal value]. SUVr, standardized uptake value ratio; MMSE, Mini Mental Score Examination; MCI, Mild Cognitive Impairment; BMI, Body Mass Index; BDI-II, Beck Depression Inventory II. Bold indicates signficant p values*.

### Association Between Sleep Parameters and Cortical Amyloid Load

**Figure 1:**
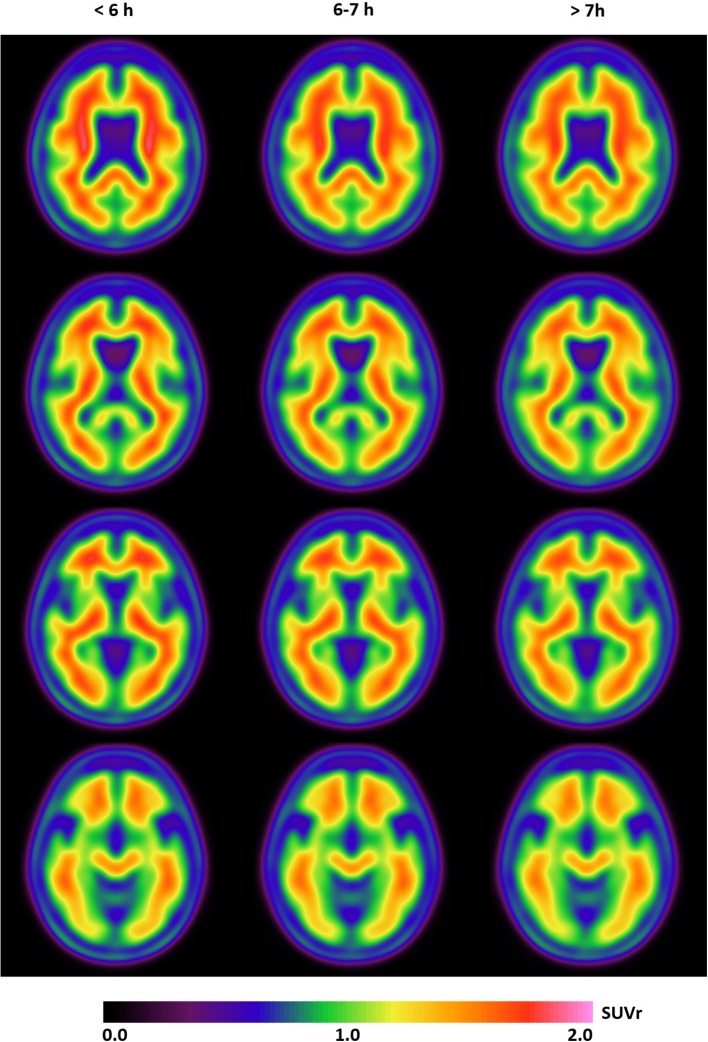
The mean SUVr (^18^F-florbetapir images) from four axial slices in participants divided according to their sleep duration (≤6, 6–7, and >7 h) show no difference in Aβ burden in these three groups.

^18^F-florbetapir**-**PET was performed within a median interval of 134 [10–360] days from the sleep evaluation. No significant relationship was found between positive amyloid PET and nighttime sleep duration [taken as a continuous variable and categorized into <6; 6–7; ≥7 h per night as previously reported (5)] before and after adjustment for APOEε4 and depressive symptoms (BDI-II score ≥12) (Table 2 and Figure 1). Similarly, no association was found between amyloid PET status and other sleep characteristics: daytime sleep duration, 24-h sleep duration, napping, sleep efficiency, EDS, insomnia symptoms, risk of sleep apnea, RLS, and clinically-defined RBD (Table 2). These results remained unchanged after excluding subjects with MCI (*n* = 36).

**Table 2:** Sleep characteristics of participants divided according to the pathological cut-off value and to tertiles for the global cortical SUVr calculated using the ^18^F-florbetapir-PET data.

**Global cortical SUVr**																
	**≤1.17** ***N*** **=** **85**		**>1.17** ***N*** **=** **58**		**Model 0**		**Model 1**		**≤1.22** ***N*** **=** **94**		**>1.22** ***N*** **=** **49**		**Model 0**		**Model 1**	
**Variable**	***n***	**%**	***N***	**%**	**OR [95% CI]**	***P***	**OR [95% CI]**	***P***	***n***	**%**	***n***	**%**	**OR [95% CI]**	***P***	**OR [95% CI]**	***P***
**Nighttime sleep duration, hours**																
<6	13	15.29	11	18.97	1	0.63	1	0.89	17	18.09	7	14.29	1	0.38	1	0.39
6–7	44	51.76	32	55.17	0.86 [0.342; 0.16]		1.01 [0.362; 0.87]		46	48.94	30	61.22	1.58 [0.594; 0.28]		2.15 [0.686; 0.87]	
≥7	28	32.94	15	25.86	0.63 [0.231; 0.75]		0.82 [0.262; 0.61]		31	32.98	12	24.49	0.94 [0.312; 0.84]		1.53 [0.425; 0.55]	
Nighttime sleep duration, hours	7 [4–10]		7 [4–9]		0.87 [0.661; 0.15]	0.33	0.89 [0.641; 0.22]	0.47	7 [4–10]		7 [4.5–9]		0.92 [0.691; 0.24]	0.60	0.98 [0.701; 0.37]	0.90
**Daytime sleep duration, minutes**																
Daytime sleep duration, *min* ^(1)^	15 [0–120]		20 [0–90]		1.00 [0.991; 0.02]	0.71	1.01 [0.991; 0.02]	0.49	15 [0–120]		20 [0–90]		1.00 [0.991; 0.02]	0.97	1.01 [0.991; 0.02]	0.55
**Naps**																
No	35	45.45	23	44.23	1	0.77	1	0.48	37	44.05	21	46.67	1	0.81	1	0.59
Yes <30 min	21	27.27	12	23.08	0.87 [0.362; 0.10]		1.81 [0.664; 0.97]		23	27.38	10	22.22	0.77 [0.311; 0.91]		1.71 [0.594; 0.95]	
Yes ≥30 min	21	27.27	17	32.69	1.23 [0.542; 0.82]		1.47 [0.573; 0.84]		24	28.57	14	31.11	1.03 [0.442; 0.40]		1.36 [0.513; 0.67]	
**Sleep efficiency (%)**																
<82.35	28	32.94	19	32.76	1	0.90	1	0.53	29	30.85	18	36.73	1	0.77	1	0.61
[82.35–93.75]	29	34.12	18	31.03	0.91 [0.402; 0.09]		0.85 [0.332; 0.15]		32	34.04	15	30.61	0.76 [0.321; 0.77]		0.67 [0.261; 0.77]	
≥93.75	28	32.94	21	36.21	1.11 [0.492; 0.49]		1.41 [0.563; 0.59]		33	35.11	16	32.65	0.78 [0.341; 0.81]		1.05 [0.402; 0.75]	
Sleep efficiency (%)^(a)^	87.5 [44.4–100]		88.9 [50-100]		1.01 [0.991; 0.04]	0.39	1.02 [0.991; 0.06]	0.12	87.5 [44.4–100]		87.5 [62.5–100]		1.01 [0.981; 0.03]	0.64	1.02 [0.991; 0.05]	0.18
**ESS score**																
<11	66	78.57	40	76.92	1	0.82	1	0.68	71	77.17	35	79.55	1	0.76	1	0.98
≥11	18	21.43	12	23.08	1.10 [0.48; 2.52]		1.22 [0.483; 0.09]		21	22.83	9	20.45	0.87 [0.362; 0.10]		1.01 [0.372; 0.77]	
ESS score^(a)^	6 [0–21]		6 [1–15]		1.01 [0.931; 0.10]	0.86	1.01 [0.911; 0.11]	0.89	6 [0–21]		6 [1–14]		0.99 [0.901; 0.08]	0.82	0.99 [0.891; 0.10]	0.84
**Insomnia severity scale**																
<8	55	65.48	36	63.16	1	0.75	1	0.44	60	65.22	31	63.27	1	0.29	1	0.12
8–14	21	25.00	17	29.82	1.24 [0.582; 0.66]		1.08 [0.422; 0.78]		22	23.91	16	32.65	1.41 [0.653; 0.06]		1.24 [0.473; 0.28]	
≥15	8	9.52	4	7.02	0.76 [0.212; 0.72]		0.38 [0.071; 0.93]		10	10.87	2	4.08	0.39 [0.081; 0.88]		0.11 [0.011; 0.11]	
Insomnia severity scale^(a)^	5 [0-23]		4 [0-23]		0.99 [0.931; 0.06]	0.81	0.95 [0.871; 0.04]	0.25	5 [0-23]		4 [0-15]		0.96 [0.901; 0.03]	0.31	0.91 [0.821; 0.00]	0.04
**Restless legs syndrome**																
No	71	84.52	51	89.47	1	0.40	1	0.41	77	82.80	45	93.75	1	0.08	1	0.07
Yes	13	15.48	6	10.53	0.64 [0.231; 0.80]		0.63 [0.211; 0.91]		16	17.20	3	6.25	0.32 [0.091; 0.16]		0.28 [0.071; 0.10]	
**Risk of sleep apnea**																
Low	39	60.00	30	61.22	1	0.89	1	0.57	44	60.27	25	60.98	1	0.94	1	0.51
High	26	40.00	19	38.78	0.95 [0.442; 0.03]		1.28 [0.543; 0.01]		29	39.73	16	39.02	0.97 [0.442; 0.13]		1.35 [0.553; 0.29]	
**Clinically-defined RBD**																
No	64	94.12	36	87.80	1	0.26	1	0.38	68	93.15	32	88.89	1	0.45	1	0.70
Yes	4	5.88	5	12.20	2.22 [0.568; 0.80]		1.98 [0.439; 0.09]		5	6.85	4	11.11	1.70 [0.436; 0.76]		1.37 [0.276; 0.95]	

(a) Continuous variables were expressed as median [minimal value-maximal value].

ESS, Epworth Severity Scale; RBD, REM sleep behavioral disorder.

Model 0: Crude association.

*Model 1: Adjustment for APOEε4 and Beck Depression Inventory-II score in two classes (score 0–11: no depressive symptoms, and score ≥ 12: moderate to severe depressive symptoms)*.

Comparison of participants subdivided in two groups according to their global cortical SUVr values in tertiles (highest tertile, SUVr >1.22, vs. the other two) showed almost similar results, with no between-group differences (Table 2).

## Discussion

Our study provides an extensive assessment of the sleep-wake profile and brain amyloid load measured with ^18^F-florbetapir-PET in a cohort of elderly subjects without dementia. No association was found between the global cortical (using the pathological cut-off of 1.17 and tertiles of the population) and nighttime sleep duration, but also with daytime sleep duration, naps, daytime sleepiness, insomnia, risk of apnea, clinically-defined RBD, and sleep efficiency in unadjusted and adjusted models for APOEε4 and depressive symptoms.

A similar key study on 62 community-dwelling older adults found that people with self-reported sleep ≤ 6 h/night had higher Aβ burden measured by ^11^C-Pittsburgh compound B (PiB) PET in cortical and precuneus areas than those with >7 h of sleep/night. Amyloid burden was intermediate in subjects with 6–7 h of sleep per night (5). These results remained significant after adjustment for potential confounders (APOEε4 carrier status, comorbidities and sleep-related treatments) (5). Despite similar self-reported assessment of sleep duration among both studies, our study did not confirm such association in unadjusted and adjusted models for APOEε4 status and depressive symptoms. Conversely, the number of participants (143 in our study vs. 62), amyloid tracer (^18^F-florbetapir vs. ^11^C-PiB), tracer uptake quantification (SUVr vs. distribution volume ratio), segmentation method (PET vs. MRI template), and cognitive profile (25.2% of subjects with MCI in our population vs. cognitively normal participants; but results from our study remained unchanged after excluding subjects with MCI) were different (5). Except for the low sample size at risk of obtaining a Type I error, other differences are less likely to explain such result discrepancies. We also reanalyzed our data using linear instead of logistic regression models, like in the previous study (5), but results remain unchanged. Also, we found no association between amyloid load in the precuneus specifically and nighttime sleep duration (data not shown).

Although the potential relationship between sleep duration and amyloid load (1, 16) is attractive in the context of amyloid clearance, sleep-wake patterns (27), orexin involvement (28), and glymphatic alterations in AD (29–31), the links between sleep and AD pathology are complex, often bidirectional and variable during disease progression (32). Extracellular amyloid accumulation could be reduced during sleep and increased during wakefulness (33). Recent studies performed in young healthy adults showed that slow-wave sleep, rather that the entire sleep period, plays a key role in regulating the cerebrospinal fluid (CSF) levels of Aβ (34). As slow waves change in amplitude, frequency and shape with aging (35), we could hypothesize that the links between sleep and production/clearance of soluble amyloid proteins are different in older individuals compared with young people. Nevertheless, our current findings do not confirm that insufficient sleep duration is a clinically significant risk factor for brain amyloid deposition in elderly subjects.

Our study also did not find any association between amyloid PET positivity and napping, EDS, and sleep efficiency. Conversely, a study with a different design (actigraphy and CSF Aβ quantification in 142 healthy volunteers) showed an association between low CSF Aβ_42_ levels and frequent napping, sleep efficiency, but again not with nighttime sleep duration (12). A cross-sectional study on 184 cognitively normal participants older than 60 years found that longer sleep latency was associated with higher levels of amyloid burden (by PET), independently of the APOEε4 status (14). Similarly, another study found that cognitively healthy adults with less adequate sleep, more sleep problems, and greater somnolence (assessed using the Sleep Scale from the Medical Outcomes Study) had higher amyloid load in angular gyrus, frontal medial orbital cortex, cingulate gyrus, and precuneus (6). However, amyloid burden was not associated with the daytime sleepiness assessed by ESS (6). On the other hand, a recent study on 283 ≥70-year-old participants without dementia found that baseline excessive daytime sleepiness (ESS score ≥10) was significantly associated with longitudinal Aβ accumulation in the anterior and posterior cingulate, precuneus and parietal regions within a mean interval between two ^11^C-PiB-PET scans of 2.2 years (36). Unfortunately, the absence of reported cross-sectional results on amyloid burden, sleep profile, particularly nighttime sleep duration and excessive daytime sleepiness, does not allow comparing our findings. Using the Berlin questionnaire, we did not find any association between amyloid PET status and the risk of sleep apnea. In contrast, several recent studies underlined the impact of self-reported clinical diagnosis of obstructive sleep apnea on the longitudinal increases in florbetapir PET uptake in normal and MCI subjects (37, 38). The mechanistic processes that link sleep apnea to accumulation of amyloid plaques and dementia need to be better assessed before proposing novel targets for intervention (37, 38).

The strengths of our study are the well-characterized population with spontaneous memory complaints but free of dementia, the standardized ^18^F-florbetapir-PET imaging with cortical SUVr measurements, the clinical face-to-face sleep interview associated with validated questionnaires, and the analysis of a large number of potential confounding factors. The absence of relationship between positive amyloid PET and nighttime sleep duration persists despite adjustment for APOEε4 and depressive symptoms. To fill the gap between cognitively normal participants and prodromal AD, subjects with memory complaints constitute a well-targeted population.

The present study also presents some limitations. The sleep profile assessment was self-reported only, based on a clinical interview and questionnaires completed by the patients with the help of a caregiver/clinical team member if required, to detail sleep characteristics and complaints at time of study instead of objective measurements, such as actigraphy or polysomnography. The self-reporting nature of the evaluation could have led to recall biases and lack of accuracy in the responses, with potential unstable sleep phenotype/complaints and sleep misperception. However, the use of self-reported questionnaires to detect individuals with sleep disturbances is particularly relevant in the clinical practice and was easier and less expensive than polysomnography in a sleep laboratory but preclude to detect sleep apneas. Finally, we are aware that small sample size is often a limitation especially when reporting negative results. However, the number of participants was more than twice that of the previous study (5). We have computed a *post-hoc* statistical power calculation between the groups in our population. With the SUVR threshold of 1.17 and the means of sleep duration of 6.83 h in subjects below 1.17 and 6.63 h in those above, common standard deviation of 1.2 and alpha risk of 0.05, 567 subjects per group (1,134 in total) would have been necessary to show significant between-group differences with a power of 0.80. To date, no study arranges data with both PET-amyloid and sleep assessments on more than 1,000 participants at-risk to develop AD.

## Conclusions

Our study failed to confirm previous findings on the association between poor sleep quantity/quality and amyloid load, despite the fairly large population, the detailed, and comprehensive sleep profile, the large number of potential confounding factors, and the cortical amyloid load measurements by ^18^F-florbetapir-PET. Conversely, our results show that the interplay between sleep and amyloid is more complex than previously described. Before developing tailored therapeutic approaches, the sleep profile of subjects with amyloid pathology at different disease stages need to be better understood.

## Data Availability

All datasets generated for this study are included in the manuscript and/or the supplementary files.

## Ethics Statement

The MAPT-AV45 study and the sleep ancillary study were approved by the Toulouse ethics committee (Comitéde Protection des Personnes- Sud-Ouest et Outre-Mer I et II). The methods were carried out in accordance with the approved guidelines. Each participant signed legal consent forms. Informed consent was obtained from all subjects.

## Author Contributions

YD and AG: drafting, revising the manuscript for content, including medical writing for content, study concept or design, interpretation of data analysis, study supervision, and coordination. L-AG, CB, and IJ: revising the manuscript for content, interpretation of data analysis, study concept or design, and statistical analysis. FB and DM-G: revising the manuscript for content, interpretation of data analysis, and acquisition of data. SN, DD, CG, KB, CM, RD, SA, BV, and PP: revising the manuscript for content and acquisition of data.

### Conflict of Interest Statement

YD has acted as a consultant for UCB Pharma, Jazz, Theranexus, Flamel, Idorsia, Takeda, Harmony Biosciences, and Bioprojet, outside the submitted work. AG has been a speaker and advisory board member for Biogen, Roche, Amgen, Novartis. AG received fundings from La Fondation Philippe Chartrier for Alzheimer projects. No conflit of interest is related to this article. The remaining authors declare that the research was conducted in the absence of any commercial or financial relationships that could be construed as a potential conflict of interest.

## References

[1] Mander BA Winer JR Jagust WJ Walker MP. Sleep: a novel mechanistic pathway, biomarker, and treatment target in the pathology of Alzheimer's disease? Trends Neurosci. (2016) 39:552–66. 10.1016/j.tins.2016.05.002 27325209 PMC4967375

[2] Lucey BP Holtzman DM. How amyloid, sleep and memory connect. Nat Neurosci. (2015) 18:933–4. 10.1038/nn.4048 26108720 PMC4770804

[3] Cedernaes J Osorio RS Varga AW Kam K Schiöth HB Benedict C. Candidate mechanisms underlying the association between sleep-wake disruptions and Alzheimer's disease. Sleep Med Rev. (2016) 31, 102–11. 10.1016/j.smrv.2016.02.002 26996255 PMC4981560

[4] Dauvilliers Y. Insomnia in patients with neurodegenerative conditions. Sleep Med. (2007) 8(Suppl 4):S27–34. 10.1016/S1389-9457(08)70006-6 18346674

[5] Spira AP Gamaldo AA An Y Wu MN Simonsick EM Bilgel M. Self-reported sleep and beta-amyloid deposition in community-dwelling older adults. JAMA Neurol. (2013) 70:1537–43. 10.1001/jamaneurol.2013.4258 24145859 PMC3918480

[6] Sprecher KE Bendlin BB Racine AM Okonkwo OC Christian BT Koscik RL. Amyloid burden is associated with self-reported sleep in nondemented late middle-aged adults. Neurobiol Aging. (2015) 36:2568–76. 10.1016/j.neurobiolaging.2015.05.004 26059712 PMC4523445

[7] Branger P Arenaza-Urquijo EM Tomadesso C Mézenge F André C de Flores R. Relationships between sleep quality and brain volume, metabolism, and amyloid deposition in late adulthood. Neurobiol Aging. (2016) 41:107–14. 10.1016/j.neurobiolaging.2016.02.009 27103523

[8] Osorio RS Ayappa I Mantua J Gumb T Varga A Mooney AM. Interaction between sleep-disordered breathing and apolipoprotein E genotype on cerebrospinal fluid biomarkers for Alzheimer's disease in cognitively normal elderly individuals. Neurobiol Aging. (2014) 35:1318–24. 10.1016/j.neurobiolaging.2013.12.030 24439479 PMC4022140

[9] Emamian F Khazaie H Tahmasian M Leschziner GD Morrell MJ Hsiung GY. The association between obstructive sleep apnea and Alzheimer's disease: a meta-analysis perspective. Front Aging Neurosci. (2016) 8:78. 10.3389/fnagi.2016.00078 27148046 PMC4828426

[10] Yaffe K Laffan AM Harrison SL Redline S Spira AP Ensrud KE. Sleep-disordered breathing, hypoxia, and risk of mild cognitive impairment and dementia in older women. JAMA. (2011) 306:613–9. 10.1001/jama.2011.1115 21828324 PMC3600944

[11] Varga AW Wohlleber ME Giménez S Romero S Alonso JF Ducca EL. Reduced slow-wave sleep is associated with high cerebrospinal fluid Abeta42 levels in cognitively normal elderly. Sleep. (2016) 39:2041–8. 10.5665/sleep.6240 27568802 PMC5070758

[12] Ju YE McLeland JS Toedebusch CD Xiong C Fagan AM Duntley SP. Sleep quality and preclinical Alzheimer disease. JAMA Neurol. (2013) 70:587–93. 10.1001/jamaneurol.2013.2334 23479184 PMC3676720

[13] Drogos LL Gill SJ Tyndall AV Raneri JK Parboosingh JS Naef A. Evidence of association between sleep quality and APOE epsilon4 in healthy older adults: a pilot study. Neurology. (2016) 87:1836–42. 10.1212/WNL.0000000000003255 27777343 PMC5089524

[14] Brown BM Rainey-Smith SR Villemagne VL Weinborn M Bucks RS Sohrabi HR. The Relationship between sleep quality and brain amyloid burden. Sleep. (2016) 39:1063–8. 10.5665/sleep.5756 27091528 PMC4835305

[15] Gabelle A Gutierrez LA Jaussent I Navucet S Grasselli C Bennys K. Excessive sleepiness and longer nighttime in bed increase the risk of cognitive decline in frail elderly subjects: the MAPT-sleep study. Front Aging Neurosci. (2017) 9:312. 10.3389/fnagi.2017.00312 29033827 PMC5625324

[16] Vellas B Carrie I Gillette-Guyonnet S Touchon J Dantoine T Dartigues JF. Mapt study: a multidomain approach for preventing Alzheimer's disease: design and baseline data. J Prevent Alzheimer's Dis. (2014) 1:13–22. 26594639 PMC4652787

[17] Payoux P Delrieu J Gallini A Adel D Salabert AS Hitzel A. Cognitive and functional patterns of nondemented subjects with equivocal visual amyloid PET findings. Euro J Nuclear Med Mol Imaging. (2015) 42:1459–68. 10.1007/s00259-015-3067-9 25952279

[18] Gillette-Guyonnet S Andrieu S Dantoine T Dartigues JF Touchon J Vellas B Commentary on A roadmap for the prevention of dementia II. Leon Thal Symposium 2008. The multidomain alzheimer preventive trial (MAPT): a new approach to the prevention of Alzheimer's disease. Alzheimer's Dementia. (2009) 5:114–21. 10.1016/j.jalz.2009.01.008 19328438

[19] CarriéIvan KanGAGillette-GuyonnetSAndrieuSDartiguesJFTouchonJ. Recruitment strategies for preventive trials. The MAPT study (MultiDomain Alzheimer Preventive Trial). J Nutrit Health Aging. (2012) 16:355–9. 10.1007/s12603-012-0046-822499458

[20] Joshi AD Pontecorvo MJ Clark CM Carpenter AP Jennings DL Sadowsky CH. Performance characteristics of amyloid PET with florbetapir F 18 in patients with alzheimer's disease and cognitively normal subjects. J Nuclear Med. (2012) 53:378–4. 10.2967/jnumed.111.090340 22331215

[21] Allen RP Picchietti DL Garcia-Borreguero D Ondo WG Walters AS Winkelman JW. Restless legs syndrome/Willis-Ekbom disease diagnostic criteria: updated International restless legs syndrome study group (IRLSSG) consensus criteria–history, rationale, description, and significance. Sleep Med. (2014) 15:860–73. 10.1016/j.sleep.2014.03.025 25023924

[22] Johns MW. A new method for measuring daytime sleepiness: the Epworth sleepiness scale. Sleep. (1991) 14:540–5. 10.1093/sleep/14.6.540 1798888

[23] Bastien CH Vallières A Morin CM. Validation of the Insomnia severity Index as an outcome measure for insomnia research. Sleep Med. (2001) 2:297–307. 10.1016/S1389-9457(00)00065-4 11438246

[24] Netzer NC Stoohs RA Netzer CM Clark K Strohl KP. Using the Berlin questionnaire to identify patients at risk for the sleep apnea syndrome. Annals Int Med. (1999) 131:485–91. 10.7326/0003-4819-131-7-199910050-00002 10507956

[25] Beck AT Ward CH Mendelson M Mock J Erbaugh J. An inventory for measuring depression. Arch Gen Psychiatry. (1961) 4:561–71. 10.1001/archpsyc.1961.01710120031004 13688369

[26] Winblad B Palmer K Kivipelto M Jelic V Fratiglioni L Wahlund LO. Mild cognitive impairment–beyond controversies, towards a consensus: report of the International working group on mild cognitive impairment. J Int Med. (2004) 256:240–6. 10.1111/j.1365-2796.2004.01380.x 15324367

[27] Lucey BP Mawuenyega KG Patterson BW Elbert DL Ovod V Kasten T. Associations between beta-amyloid kinetics and the beta-amyloid diurnal pattern in the central nervous system. JAMA Neurol. (2017) 74:207–15. 10.1001/jamaneurol.2016.4202 27992627 PMC5305432

[28] Gabelle A Jaussent I Hirtz C Vialaret J Navucet S Grasselli C. Cerebrospinal fluid levels of orexin-A and histamine, and sleep profile within the Alzheimer process. Neurobiol Aging. (2017) 53:59–66. 10.1016/j.neurobiolaging.2017.01.011 28235679

[29] Nedergaard M Goldman SA. Brain drain. Sci Am. (2016) 314:44–9. 10.1038/scientificamerican0316-44 27066643 PMC5347443

[30] Jessen NA Munk AS Lundgaard I Nedergaard M. The glymphatic system: a beginner's guide. Neurochem Res. (2015) 40:2583–99. 10.1007/s11064-015-1581-6 25947369 PMC4636982

[31] Peng W Achariyar TM Li B Liao Y Mestre H Hitomi E. Suppression of glymphatic fluid transport in a mouse model of Alzheimer's disease. Neurobiol Dis. (2016) 93:215–25. 10.1016/j.nbd.2016.05.015 27234656 PMC4980916

[32] Suh SW Han JW Lee JR Byun S Kwon SJ Oh SH. Sleep and cognitive decline: a prospective nondemented elderly cohort study. Annals Neurol. (2018) 83:472–82. 10.1002/ana.25166 29394505

[33] Lucey BP Hicks TJ McLeland JS Toedebusch CD Boyd J Elbert DL Effect of sleep on overnight CSF amyloid-beta kinetics. Annals Neurol. (2017) 83:197–204. 10.1002/ana.25117 PMC5876097 29220873

[34] Ju YS Ooms SJ Sutphen C Macauley SL Zangrilli MA Jerome G. Slow wave sleep disruption increases cerebrospinal fluid amyloid-beta levels. Brain. (2017) 140:2104–11. 10.1093/brain/awx148 28899014 PMC5790144

[35] Skeldon AC Derks G Dijk DJ002E Modelling changes in sleep timing and duration across the lifespan: changes in circadian rhythmicity or sleep homeostasis? Sleep Med Rev. (2016) 28:96–107. 10.1016/j.smrv.2015.05.011 26545247

[36] Carvalho DZ St Louis EK Knopman DS Boeve BF Lowe VJ Roberts RO. Association of excessive daytime sleepiness with longitudinal beta-amyloid accumulation in elderly persons without dementia. JAMA Neurol. (2018) 75:672–80. 10.1001/jamaneurol.2018.0049 29532057 PMC5885188

[37] Bubu OM Pirraglia E Andrade AG Sharma RA Gimenez-Badia S Umasabor-Bubu OQ. Obstructive sleep apnea and longitudinal Alzheimer's disease biomarker changes. Sleep. (2019) 42:zsz048. 10.1093/sleep/zsz048 30794315 PMC6765111

[38] Gosselin N Baril AA Osorio RS Kaminska M Carrier J. Obstructive sleep apnea and the risk of cognitive decline in older adults. Am J Respir Crit Care Med. (2019) 199:142–8 10.1164/rccm.201801-0204PP 30113864 PMC6943882

